# Complete genome sequence and genomic characterization of *Microcystis panniformis* FACHB 1757 by third-generation sequencing

**DOI:** 10.1186/s40793-016-0130-5

**Published:** 2016-01-28

**Authors:** Jun-Yi Zhang, Rui Guan, Hu-Jun Zhang, Hua Li, Peng Xiao, Gong-Liang Yu, Lei Du, De-Min Cao, Bing-Chuan Zhu, Ren-Hui Li, Zu-Hong Lu

**Affiliations:** State Key Laboratory of Bioelectronics, School of Biological Sciences and Medical Engineering, Southeast University, Nanjing, 210096 China; Wuxi Environmental Monitoring Center, Wuxi, 214121 China; Institute of Hydrobiology, The Chinese Academy of Sciences, Wuhan, Hubei 430072 China; Shenzhen Key Laboratory for Marine Bio-resource and Eco-environment, College of Life Sciences, Shenzhen University, Shenzhen, 518060 China; Nextomics Biosciences Co., Ltd., Wuhan, Hubei 430072 China; Department of Biomedical Engineering, College of Engineering, Peking University, Beijing, 100871 China

**Keywords:** *Microcystis panniformis* FACHB1757, *Microcystis*, Lake Taihu, Water bloom, Third-generation sequencing, Comparative genomics

## Abstract

The cyanobacterial genus *Microcystis* is well known as the main group that forms harmful blooms in water. A strain of *Microcystis*, *M. panniformis* FACHB1757, was isolated from Meiliang Bay of Lake Taihu in August 2011. The whole genome was sequenced using PacBio RS II sequencer with 48-fold coverage. The complete genome sequence with no gaps contained a 5,686,839 bp chromosome and a 38,683 bp plasmid, which coded for 6,519 and 49 proteins, respectively. Comparison with strains of *M. aeruginosa* and some other water bloom-forming cyanobacterial species revealed large-scale structure rearrangement and length variation at the genome level along with 36 genomic islands annotated genome-wide, which demonstrates high plasticity of the *M. panniformis* FACHB1757 genome and reveals that *Microcystis* has a flexible genome evolution.

**Electronic supplementary material:**

The online version of this article (doi:10.1186/s40793-016-0130-5) contains supplementary material, which is available to authorized users.

## Introduction

The massive development of bloom-forming cyanobacteria is causing problems in eutrophic water bodies worldwide. Among the cyanobacteria, *Microcystis* is perhaps the most notorious. Many *Microcystis* species have been reported to be able to produce microcystins [1–4], which threaten many aquatic ecosystems and cause serious and occasionally fatal human liver, digestive, neurological, and skin diseases [5–7].

*Microcystis* is a genus of unicellular colony-forming cyanobacteria whose taxonomy is still unclear [8]. Although morphological criteria have been proposed to distinguish *Microcystis* species from field samples, such criteria have long been questioned for use in species identification within the genus [9]. Several studies attempted to reconcile molecular and morphological taxonomy in *Microcystis* [9–14], and a morphology-based taxonomic system has been dominantly used. *Microcystis**panniformis* was first reported in 2002 and was morphologically described as having flattened, irregular, monolayer colonies with small holes inside and later disintegrated into small pieces [15]. Since the *M. panniformis* strain SPC 702 was successfully isolated from Lago das Garças, São Paulo in 1999, studies addressing different aspects of this species have been performed [16–25]. In China, *M. panniformis* was reported as a newly recorded species in 2012 [26], and one strain (FACHB1757) was isolated from Lake Taihu. *Microcystis**panniformis* was originally thought to only be distributed in tropical regions, but we showed that this species has invaded the subtropical regions with a monsoon climate [26]. Global expansion of harmful cyanobacteria has been thought to be linked to climate changes, particularly increasing amounts of atmospheric CO_2_ and surface temperature, which may promote *Microcystis* growth and enhance the potential for bloom occurrence [27–29]. Therefore, a deeper understanding of the ecology and physiology of *M. panniformis*FACHB1757 by obtaining a robust genome reference may provide insight into the expansion and invasion mechanisms of *Microcystis*.

## Organism information

### Classification and features

**Fig. 1:**
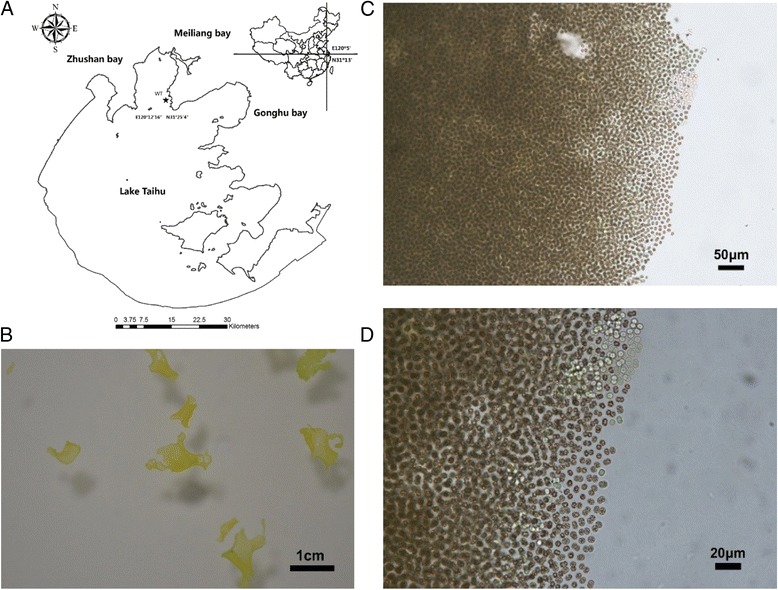
Strain collection location and photomicrographs of *M. panniformis* FACHB1757. The strain was originally isolated from Meiliang Bay of Lake Taihu in August 2011 and deposited in the Freshwater Algae Culture Collection at the Institute of Hydrobiology (FACHB-collection, China) with the unique identifier FACHB1757 in 2012. **a** The precise position of the isolated sample is indicated by a star; WT means Wutang station in Lake Taihu. **b** The morphology of the strain colonies in the white disk, which were collected directly from the water surface using a plastic bucket (on September 15, 2013 in Meiliang Bay, photo with a Nikon D7000). **c**, **d** Flat colonies with small holes as viewed under an optical microscope

A water bloom sample was collected directly from the water surface using a plastic bucket in Meiliang Bay of Lake Taihu in August 2011 (Fig. 1a). Lake Taihu (E 30°56′–31°33′,N 119°54′–120°36′), the third largest freshwater lake in China, is located in the south of the Yangtze River Delta. The total area of the lake is 2338 km^2^, with an average depth of 2 m and total capacity of 47.6 × 10^8^ m^3^. Lake Taihu is situated in the subtropical zone with a humid and semi-humid monsoon climate, and has suffered from severe eutrophication over the past three decades. Meiliang Bay is located in the northern part of Lake Taihu (Fig. 1a), which has a surface area of 100 km^2^, depth of 1.8–2.3 m, and is currently hypereutrophic [30].

Some *Microcystis* colonies in the sample disintegrated during the sample collection process; thus, only those macroscopic colonies with significant monolayer were collected with 3-ml pipets (BD Falcon, USA), and transferred into 50-ml centrifuge tubes (Corning, USA), and immediately shipped to the laboratory. Finally, macroscopic colonies that had flattened irregular up to monolayers with small holes (in old colonies) were identified as *M. panniformis* by examination under an optical microscope. *Microcystis**panniformis*FACHB1757 was obtained, and this strain was then stored at the Freshwater Algae Culture Collection at the Institute of Hydrobiology, Chinese Academy of Sciences.

**Fig. 2:**
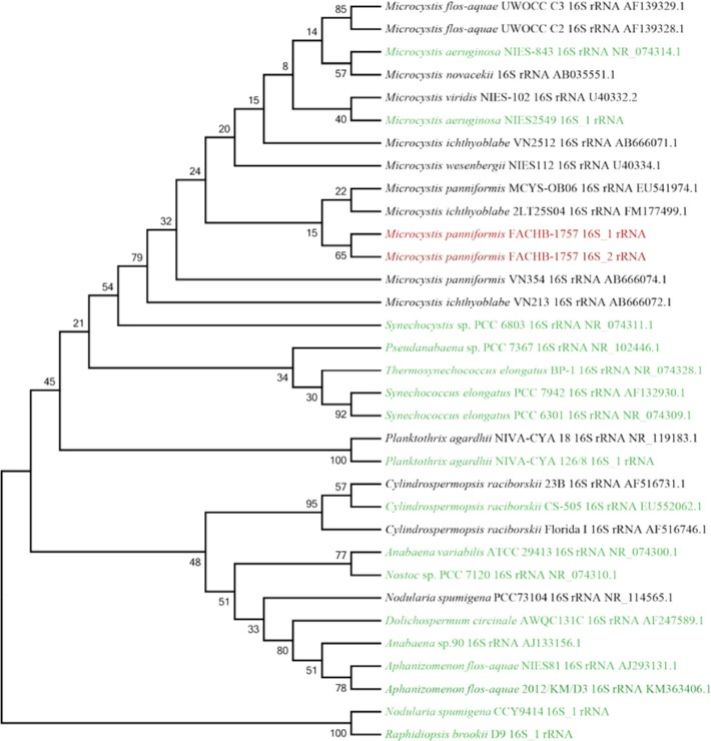
Phylogenetic tree showing the position of *M. panniformis* FACHB1757. The dendrogram is based on the 16S ribosome RNA complete sequence of *M. panniformis* FACHB1757, *M. aeruginosa* NIES843, *M. aeruginosa* PCC7806, *M. aeruginosa* NIES 2549, and representatives of other cyanobacterial genera (*Synechocystis*, *Pseudanabaena*, *Synechococcu*s, *Thermosynechococcu*s, *Planktothrix*, *Dolichospermum*, *Anabaena*, *Cylindrospermopsis*, *Nodularia*, *Nostoc*, *Aphanizomenon*, *Raphidiopsis*) downloaded from NCBI (sequences without accession numbers were extracted from annotation files of the corresponding genomes) using the neighbor-joining algorithm with 100 bootstrap replications using MEGA6. A bootstrap consensus tree was constructed and is shown. The two copies of 16S rRNA sequences of *M. panniformis* FACHB1757 are labeled in red. The relationship between *M. panniformis* FACHB1757 and other important algae species in *Cyanophyceae* are demonstrated. Species colored in green have whole genome data available in NCBI

The general characteristics of *M. panniformis*FACHB1757 are summarized in Table 1, and a phylogenetic tree based on 16S rRNA sequences is shown in Fig. 2. The spherical cells are estimated with a diameter of 2.6 to 6.8 μm (mean 4.7 μm), be densely agglomerated, and form irregular colonies with small holes. The young stages formed small clusters of cells, which were flat or circular in outline, sometimes spheroidal, and with or without an internal hollow. The old stages formed colonies with small holes, which later disintegrated into small groups. The mucilage (margin of colonies) was diffuse, and cells did not overlap. The margin of the colonies was smooth or (in old colonies) irregular. Cell density was regular and evenly agglomerated, sometimes in indistinct rows. Diagnostic characteristics included flat colonies with small holes, toxicity, homogeneously arranged cells, and life cycle was characterized by distinct benthic and planktonic phases [15, 31]. The distribution was tropical, and this is likely a pantropical species (e.g., S. Africa, N. Australia, S. America, Africa, China, Vietnam and New Zealand) [13, 15, 16, 26, 31, 32].

**Table 1:** Classification and general features of *M. panniformis* FACHB1757 according to the MIGS recommendations [69]

MIGS ID	Property	Term	Evidence code^a^
	Classification	Domain *Bacteria*	TAS [70]
		Phylum *Cyanobacteria*	TAS [71, 72]
		Class *Oscillatoriophycideae*	TAS [73]
		Order *Chroococcales*	TAS [73, 74]
		Family *Microcystaceae*	TAS [74]
		Genus *Microcystis*	TAS [71, 75]
		Species *M. panniformis*	TAS [15, 31]
		Strain: *M. panniformis* FACHB1757	TAS [26]
	Gram stain	Gram-negative	TAS [76]
	Cell shape	Spherical cells	TAS [15]
	Motility	Non-motile	NAS
	Sporulation	None	TAS [76]
	Temperature range	Mesophile	NAS
	Optimum temperature	29.5 °C	IDA
	pH range; Optimum	pH 7.50-9.21; pH 8.33	IDA
	Carbon source	Autotroph, heterotroph	NAS
MIGS-6	Habitat	Fresh water	NAS
MIGS-6.3	Salinity	1.0 % (maximum)	IDA
MIGS-22	Oxygen requirement	Aerobic	NAS
MIGS-15	Biotic relationship	Free-living	NAS
MIGS-14	Pathogenicity	Microcystins (MCY)	TAS [25, 77]
MIGS-4	Geographic location	Isolated Lake Taihu, China	IDA
MIGS-5	Sample collection	August, 2015	IDA
MIGS-4.1	Latitude	31.421 N	IDA
MIGS-4.2	Longitude	120.201E	IDA
MIGS-4.3	Depth	Surface 0.5 m	IDA
MIGS-4.3	Altitude	11 m	IDA

^a^Evidence codes - *IDA* Inferred from Direct Assay, *TAS* Traceable Author Statement (i.e., a direct report exists in the literature), *NAS* Non-traceable Author Statement (i.e., not directly observed for the living, isolated sample, but based on a generally accepted property for the species, or anecdotal evidence). These evidence codes are from the Gene Ontology project [78]

#### Phylogenetic analysis

**Fig. 3:**
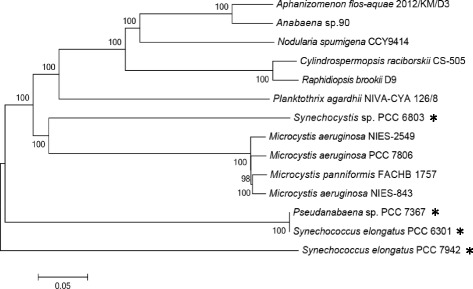
Phylogenetic tree of water bloom-forming cyanobacterial species and representative cyanobacteria. The nucleotide divergence tree was constructed using the neighbor-joining algorithm based on 691 sequences of single-copy gene families annotated by OrthoMCL with 100 bootstrap replicates. The representative cyanobacteria that cannot form water blooms are indicated with an asterisk

Whole genome comparative analysis between *M. panniformis*FACHB1757 and 13 other cyanobacterial species was performed. General information of related genome data is shown in Table S1 (Additional file 1), and all data sets were downloaded from NCBI. The main water bloom-forming cyanobacterial species in freshwater and brackish water worldwide, particularly those in the Lake Taihu region, were included. Unicellular colony-forming *Microcystis* and filamentous heterocystous *Dolichospermum* (formerly known as the planktonic *Anabaena*) were the main components of cyanobacterial blooms in Lake Taihu [33]. The *Aphanizomenon*, *Pseudanabaena*, *Cylindrospermopsis*, *Raphidiopsis*, *Planktothrix*, *Synechocystis*, and *Synechococcus* species occurred as dominant species or accompanying species in blooms of Lake Taihu (including Lake Wuli) across different seasons. Among the 14 genome sequences, 691 single-copy gene families were annotated by OrthoMCL (version 2.0.9) [34], and MEGA6 [35] was used to construct a phylogenetic tree based on these sequences (Fig. 3).

The phylogenetic tree shows that *M. panniformis*FACHB1757 and *M. aeruginosa*NIES843 shared a significantly high similarity, and there was no clear division between *M. panniformis* and *M. aeruginosa* strains in the phylogenetic tree. The *Microcystis* lineage is distinct from the lineage that contains the unicellular *Synechocystis*, *Synechococcus**,* and other multicellular cyanobacteria. Furthermore, the *Synechocystis* sp. PCC 6803 genome is more closely related to *Microcystis* than other strains. This result is congruent with previously published results based on 16S rRNA sequences [36–39]. Topological relationships between species in the phylogenetic tree based on single-copy gene families were generally consistent with the phylogenetic tree based on 16S rRNA sequences (Fig. 2).

Although *Microcystis* can be identified based on 16S rRNA and single-copy gene families sequences at the genus level, taxonomy of *Microcystis* at the species level was controversial in the past few decades, and five species have even been unified into a single species [13]. 16S rRNA sequence estimation can be ambiguous when analyzing certain *Microcystis* species with distinct morphologies, as occurred when analyzing *M. panniformis* and *M. ichthyobabe* (Fig. 2). Therefore, the whole reference genome sequence data was expected to play a crucial role in species classification of *Microcystis*. However, the currently available cyanobacterial genome sequences are highly limited. Only three *Microcystis* strains with complete genomic sequences are available, including *M. aeruginosa*NIES843 and NIES2549, and *M. panniformis*FACHB1757 reported here. Furthermore, the further species concepts and more useful molecular approaches should be proposed to classify the species/strain divergences in *Microcystis* [40, 41].

## Genome sequencing information

### Genome project history

*Microcystis**panniformis*FACHB1757 was selected for sequencing because of its obvious morphological characteristics; in particular, the macroscopic colonies with significant monolayer can even exceed 30 mm during the summer and early autumn in Lake Taihu. More importantly, until recently, only complete genomes of *M. aeruginosa* strains (including strains NIES843 and NIES2549) have been published. The complete genome sequence of *M. panniformis*FACHB1757 would only be the third for *Microcystis*. The sample information for *M. panniformis*FACHB1757 is available in NCBI under BioSample ID SAMN03392520. A DNA library with an insert size of 10 Kb was constructed, and the whole genome was sequenced to 48-fold coverage. The completed genome sequence was assembled and uploaded to GenBank under accession number. CP011339. Project details were deposited to NCBI BioProject PRJNA277430. A summary of the project information can be found in Table 2.

**Table 2:** Project information

MIGS ID	Property	Term
MIGS-31	Finishing quality	Complete
MIGS-28	Libraries used	2 PacBio SMRT cells
MIGS-29	Sequencing platforms	PacBio RSII
MIGS-31.2	Fold coverage	43.39
MIGS-30	Assemblers	HGAP 2.2.3
MIGS-32	Gene calling method	RAST
	Locus Tag	VL20
	GenBank ID	CP011339
	GenBank Date of Release	August 11, 2015
	GOLD ID	Gp0111943
	BIOPROJECT	PRJNA277430
MIGS-13	Source Material Identifier	FACHB1757
	Project relevance	Environmental

### Growth conditions and genomic DNA preparation

*Microcystis**panniformis*FACHB1757 colonies collected from the field were grown in MA medium [42] and incubated in 24-well culture plates for 4 wk. Then, floating colonies were transferred to the capped tubes that contained 5 ml of MA culture medium to finally form a unialgal culture. All cultures were grown at 25 ± 1 °C with a 12 h light/12 h dark cycle under a photon irradiance of 25 μmol photons/(m^2^ · sec) provided by daylight fluorescent lamps. Total genomic DNA of *M. panniformis*FACHB1757 was extracted using a commercial DNA isolation kit (DNeasy^®^ Plant Mini Kit, Qiagen, USA) following the manufacturer’s instructions, and analyzed by micro-volume fluorescence detection (NanoDrop^™^ 8000 Spectrophotometer, Thermo Scientific, USA) and electrophoresis in 0.8 % agarose gel stained with ethidium bromide. The isolated DNA was eluted with 50 μl of the elution buffer from the commercial kit and then stored at −20 °C until subsequent analyses.

### Genome sequencing and assembly

First, the genome was surveyed using an Illumina Hiseq sequencer to detect the purity of the cultured unialgal strain. The insert size of the next generation pair-end library was 100 bp, and 1 Gbp raw data was produced in total. All reads were mapped to the *M. aeruginosa*NIES843 reference complete genome, and more than 80 % of reads matched well. Subsequently, the genome was sequenced using PacBio RS II. Genomic DNA was sheared by Covaris S220 g-TUBE. A 10 Kb library was constructed using a PacBio template prep kit and sequenced using the PacBio SMRT platform. In total, two SMRT cells were run, and 303 megabase pair raw data was obtained. After filtering, the mean read length was 7143 bp with a quality of 0.84, and the longest read was 31,225 bp. HGAP (version 2.2.3) was used for genome assembly. Long reads were chosen as seeds, and the other reads were mapped to the seeds using Blasr (version 1.3.1.132871) [43] for error correction. After alignment, the accuracy of seed sequences were optimized to 99 % to meet the requirements of the Sanger assembly software. There was a total of 128 Mbp of high quality long seed reads, which had an average length of 7898 bp. Celera Assembler (version 8.1) [44] was then used to assemble the seed reads into contigs and Quiver [45] was used for second error correction. Contigs were assembled into the final complete genome sequence using minimus2 in AMOS (version 3.1.0). The final genome consisted of a complete circular 5,686,839 bp chromosome with a GC content of 42.35 % and a 38,683 bp plasmid with a 43.97 % GC content. Sequencing depths were 44.85 and 128.42, respectively.

### Genome annotation

TRs were predicted by Tandem Repeat Finder (version 4.07b) [46] and Microsatellite identification tool (version 1.0), which can both identify perfect and compound micro-satellites. Prediction and annotation of the genome were done using the RAST server (version 2.0) [47]. RAST integrated tRNAscan-SE, and the *search_for_rnas* tool was used to call RNA genes across the chromosome. For gene estimation, GLIMMER2 was used to represent putative genes. Subsequently, a similar search was performed against FIGfams to identify the determined genes and annotate their functions. Moreover, all putative protein-coding genes were assigned to a category using databases including Clusters of Orthologous Groups (COG), Gene Ontology (GO), Kyoto Encyclopedia of Genes and Genomes (KEGG), Swiss-Prot, and Non-Redundant Protein Database.

## Genome properties

**Fig. 4:**
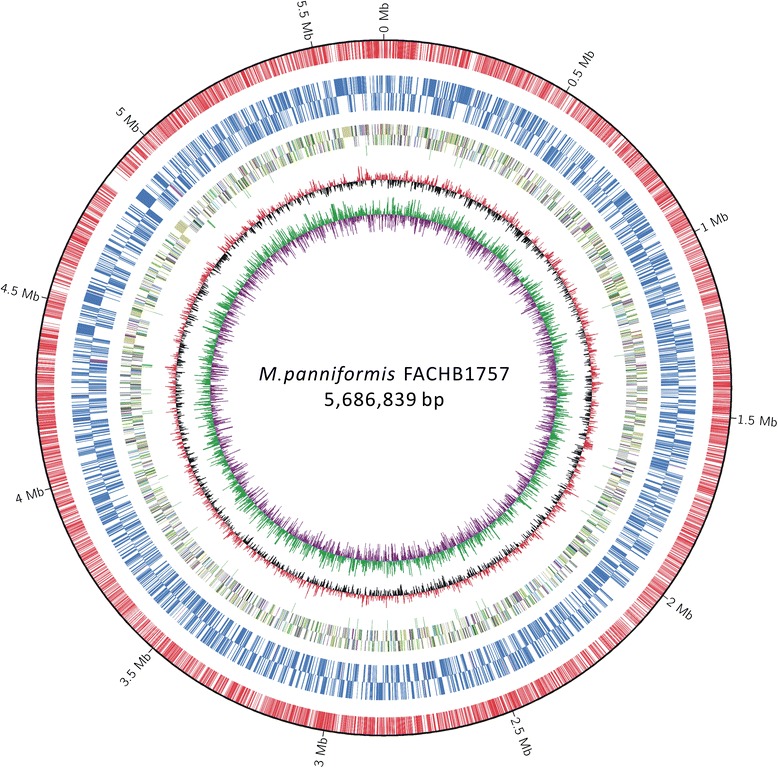
Schematic representation of the circular chromosome of *M. panniformis* FACHB1757. The scales indicate location in Mbp, starting with the initial coding region. Using Circos integrated the gene prediction results of COG function annotation, methylated modification, and some other information. From inner to outer circles: the first circle shows the GC skew (in purple and green), and the value is plotted as the deviation from the average GC skew of the entire chromosome sequence. The bars in the second circle (in black and red) represent the GC content, which is plotted using a 10-Kb sliding window. Positions of tRNA and rRNA are marked by green bars in the third circle. Bars in the fourth and fifth circle are colored according to COG function categories of CDS; the fourth is a backward strand and fifth is a forward strand. The sixth and seventh circles indicate m4C and m6A sites in CDS/rRNA/tRNA regions (in blue bars); the sixth circle is a backward strand and the seventh circle is a forward strand. In the eighth circle, red bars show the m4C and m6A sites in intergenic regions

The genome assembly contained a complete circular chromosome sequence (5.69 M) and a plasmid (38.68 K). The schematic representation of the circular chromosome of *M. panniformis*FACHB1757 was showed in Fig. 4. Related genome assembly and annotation information can be found in Table 3. Nucleotide homology search of *M. panniformis*FACHB1757 and *M. aeruginosa*NIES843 genomes was conducted by BLAST, and similarity between the two species was 83.82 % (Additional file 1: Figure S1). A total of 1944 TRs were found in the genome, including 27 microsatellites, 1742 mini-satellites, and 176 satellites. Genome statistics are shown in Table 4. In total, there were 6567 genes, which included 48 RNA genes and 6519 protein-coding genes. Among the 6519 proteins, most contained around 100 amino acids (Additional file 1: Figure S2), and by compared with function databases mentioned above, 60.15 % of them were determined to have specific functions. There were 42 tRNA genes, and two copies of the rRNA gene cluster were found in the same direction. Function assignments of 6519 putative protein-coding genes were searched against several frequently used databases mentioned above; 3260 genes were assigned to COGs, of which 235 participated in signal transduction. Search of Pfam omains detected 3997 candidates. According to the subsystem classification results processed by RAST, 72 % of determined genes belong to specific subsystems, and the distribution of each category is demonstrated in (Additional file 1: Figure S3). The result of COG function annotation is shown in Table 4, and details of each COG cluster can be found in Additional file 2. The genes assigned to GO categories by InterProScane (version 5.4-47.0) [48] were classified into cellular components, molecular functions, and biological processes. Genes distributed in each category and their functions are shown in (Additional file 1: Figure S4). In the GO data, 309 signal function-related genes were found. KEGG matched 897 functional genes to related systems, as shown in (Additional file 1: Figure S5). Final gross function annotation outcomes are provided in (Additional file 1: Table S2).

**Table 3:** Genome statistics

Attribute	Value	% of Total
Genome size (bp)	5,686,839	100.00
DNA coding (bp)	4,616,631	81.18
DNA G + C (bp)	2,408,639	42.35
DNA scaffolds	1	100.00
Total genes	6,567	100.00
Protein coding genes	6,519	99.27
RNA genes	48	0.73
rRNA genes	6	0.09
tRNA genes	42	0.64
Pseudo genes	-	-
Genes in internal clusters	-	-
Genes with function prediction	3,921	100.00
Genes assigned to COGs	3,373	86.02
Genes with Pfam domains	2,067	52.72
Genes with signal peptides	309	7.88
CRISPR repeats	3	-
Genes with transmembrane helices	-	-

**Table 4:** Number of genes associated with general COG functional categories

Code	Value	% Age	Description
J	165	2.51	Translation, ribosomal structure and biogenesis
A	0	0.00	RNA processing and modification
K	131	1.99	Transcription
L	620	9.44	Replication, recombination and repair
B	1	0.02	Chromatin structure and dynamics
D	47	0.72	Cell cycle control, Cell division, chromosome partitioning
V	67	1.02	Defense mechanisms
T	138	2.10	Signal transduction mechanisms
M	203	3.09	Cell wall/membrane biogenesis
N	30	0.46	Cell motility
U	52	0.79	Intracellular trafficking and secretion
O	152	2.31	Posttranslational modification, protein turnover, chaperones
C	185	2.82	Energy production and conversion
G	131	1.99	Carbohydrate transport and metabolism
E	215	3.27	Amino acid transport and metabolism
F	62	0.94	Nucleotide transport and metabolism
H	146	2.22	Coenzyme transport and metabolism
I	65	0.99	Lipid transport and metabolism
P	188	2.86	Inorganic ion transport and metabolism
Q	126	1.92	Secondary metabolites biosynthesis, transport and catabolism
R	535	8.14	General function prediction only
S	483	7.35	Function unknown
-	2,826	43.02	Not in COGs

The total is based on the total number of protein-coding genes in the genome

## Insights from the genome sequence

### Comparative *Microcystis* species genomes

#### Gene ortholog analysis

**Fig. 5:**
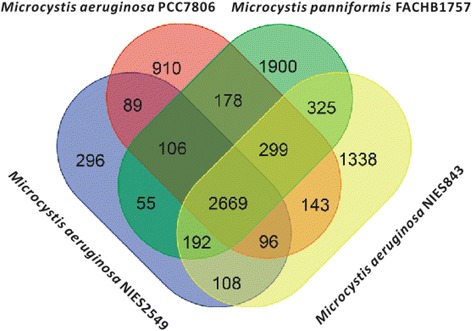
Venn diagram of gene numbers of four *Microcystis* species. Less than half of all genes were found in all four species

Genes of four *Microcystis* species were compared (Fig. 5), and 2669 highly conserved orthologous genes were shared, which are representative of the core genome. Moreover, each genome had strain-specific genes, which varied from 296 to 1900. The *M. aeruginosa*NIES2549 genome, which has 1388 unique genes, is 1.5 Mbp smaller than that of *M. aeruginosa*NIES843, which only has 296 unique genes (*M. aeruginosa*NIES843 has 1388). *Microcystis**panniformis*FACHB1757 was shown to have 1900 specific genes, which was the greatest amount among the four strains, even though its genome was not the longest.

#### Secondary metabolite gene clusters

Microcystin was reported to enhance colony formation in *Microcystis* spp. and plays a key role in the persistence of their colonies and the dominance of *Microcystis* [49]. As in *M. aeruginosa*NIES843 and *M. aeruginosa*PCC7806 genomes, the microcystin synthetase gene cluster (*mcyA-J*) was highly conserved in *M. panniformis*FACHB1757 from coordinates 3,496,704 to 3,541,027. Additionally, the distinct thioesterase type II coding gene *mcyT,* which occurs in toxic strains, and 4-PPT transferase (4-PPTase) were both located far from the *mcy* gene cluster at coordinates 869,702 to 869,286 and 915,377 to 916,039, respectively, which are similar to the distributions observed in *M. aeruginosa*NIES843. Notably, there was an absence of *mcnA* and *mcnB* in the *M. panniformis*FACHB1757 chromosome. *mcnA* codes polyketide biosynthesis proteins, and *mcnB* is the first open reading frame of mamestra configurata nucleopolyhedrovirus B. Together with *mcnC* and *mcnE*, these four genes compose the cyanopeptolin synthesis gene cluster. *mcnD* was not found in the *M. panniformis*FACHB1757 genome; thus, the cyanopeptolin produced was non-halogenated and identical to that of *M. aeruginosa*NIES843 and PCC7806. Toxins may contribute to the adaptation of this strain to its specific ecological niche in eutrophic waters of tropical and subtropical zones. In addition, a putative polyketide synthase gene cluster, which may encode additional small polypeptides found in *M. aeruginosa*NIES843 (coordinates 2,508,556–2,513,289), was detected in *M. panniformis*FACHB1757 at coordinates 4,425,371 to 4,430,104. The change in location of the genes mentioned above reflected the extensive structural variation between *M. panniformis*FACHB1757 and *M. aeruginosa*NIES843.

#### Conserved gene clusters

Four functional clusters of conserved genes related to microcystin synthesis, colony formation, photo-regulation, and nutrient assimilation were also compared among the four *Microcystis* strains. In the microcystin synthesis gene cluster, the *mcy* and *mcn* gene clusters were not found in *M. aeruginosa*NIES2549. This is consistent with the results of a previous study, which showed that *M. aeruginosa*NIES2549 is a nontoxic strain [50]. With regard to colony formation, *M. aeruginosa*, *M. wesenbergii,* and *M. panniformis* all have typical macroscopic colony structure when observed by naked eye in Lake Taihu during summer and autumn water blooms. *Microcystis**panniformis* seems to be the largest, and can even have more than 30 mm colonies. Polysaccharides and microcystin play important roles in the process of *Microcystis* colony formation. The maximum EPS content was found in *M. wesenbergii* and *M. aeruginosa*, which are not the largest and are only approximately 100 μm [51], but positive correlations between EPS and *Microcystis* colony size in cultures were supported by previous studies [52–54]. *mrpC* and *epsL* were absent from all four strains, and only *M. aeruginosa*NIES843 contained *cpsF*, although *tagH*, *capD*, *csaB*, and *rfbB* were conserved in all four strains. Furthermore, *mvn* codes for a lectin in *M. panniformis*FACHB1757 and *M. aeruginosa*PCC7806, which specifically binds to a sugar moiety present on the surface of *Microcystis* cells. Additionally, a binding partner of MVN was identified in the lipopolysaccharide fraction of *M. aeruginosa*PCC7806, which involved in the *Microcystis* colony formation [55]. Together, the toxin-, EPS-, and lectin-related genes may explain the reason why *M. panniformis*FACHB1757 usually aggregates and produces a larger colony in Lake Taihu during water blooms. In the photo-regulation cluster, *psb, apc* and *gvp* with the exception of *gvpC* were all detected. It is interesting that *gvpC* is absent from *M. panniformis*FACHB1757, because this gene encodes GvpC, which is a highly conserved expressed protein in some genera that is closely related to gas vesicles [56–58]. Genes related to nutrient assimilation include *ntc*, *pst*, and *sph* clusters. *ntcB*, *pstA*, *pstB1*, *pstB2*, and *pstC* were only absent from *M. aeruginosa*PCC7806 among the four strains, which may be accounted for by the incompleteness of the strain’s genome. Detailed information about function and coordinates of each gene are shown in (Additional file 1: Table S3).

#### Genome structure and constitution comparison

The genomes of *M. aeruginosa*NIES843 and *M. aeruginosa*NIES2549 have no plasmids, whereas a 38 Kb plasmid with a 43.97 % GC content was detected in *M. panniformis*FACHB1757 in this study. The stable presence of plasmids may play an important role in some *Microcystis* obtaining competitive advantages [59–61]. *Microcystis aeruginosa*NIES-843 is the first strain of the genus *Microcystis* to be sequenced for its complete genome with the ABI 3770xl sequencer. Since then, the second completed *Microcystis* genome (of *M. aeruginosa*NIES-2549) was released on the April 29, 2015. Thus, the whole genome at the nucleic acid level was compared between *M. panniformis*FACHB 1757 and *M. aeruginosa*NIES-843. Mauve, which was designed for identification and alignment of conserved genomic sequences with rearrangements and horizontal transfer, was used to conduct comparative genomic sequence analysis [62]. As shown in (Additional file 1: Figure S1), *M. panniformis*FACHB1757 underwent extensive chromosome structure rearrangement, which indicates that *Microcystis* genomes are highly plastic [36].

### Self-defense system

#### Restriction modification system

Comparison with REBASE [63], a restriction enzyme database containing information about restriction enzymes, revealed that DNA methyltransferases and related proteins are involved in the biological process of R–M, and 277 restriction enzymes were found. Detailed classification revealed that 12 and 130 enzymes belonged to type I and type II systems, respectively, which together represented 46.93 % of all enzymes, and are categories of rapidly evolving genes [64]. Sixty-three, 10, and 2 enzymes, respectively, belonged to type IIG, type III, and type IV systems, and one control protein restriction enzyme and 58 unknown enzymes were also found.

#### Methylation modification analysis

It is widely thought that methylation modification is associated with R-M systems and participates in self-defense against foreign genome invasion. Genome methylation modification and methyl-transferase recognition sequence motifs were analyzed using SMRT (version 2.3.0). In the chromosome, 3204 m4C (N^4^-methylcytosine), 9,758 m6A (N^6^-methyladenine), and 31,845 other modified bases were marked as modified (details are available in Additional file 3). Corresponding motif information is included in Table 5.

**Table 5:** Sequence structure and general information of motifs in the whole genome

Motif	Modified position	Type	Motifs detected	# of motifs detected	# of motifs in genome	Mean QV	Mean motif coverage	Partner motif
GATC	2	m6A	78.55 %	37,874	48,218	45.44	22.91	GATC
GAATTC	3	m6A	74.54 %	1938	2,600	44.25	22.53	GAATTC
GCTGDAG	6	m6A	73.70 %	995	1,350	43.72	22.95	-
GGTGGA	6	m6A	70.96 %	1935	2,727	43.22	22.81	-
GACGNAC	6	m6A	70.26 %	723	1,029	42.58	23.11	-
ACCACC	4	m6A	69.67 %	2410	3,459	41.91	22.82	-
CAAGNNNNNNTTTC	3	m6A	69.02 %	176	255	41.45	21.48	-
GATATC	2	m6A	67.42 %	2055	3,048	42.29	23.09	GATATC
MCGRAG	5	m6A	52.23 %	3390	6,491	41.64	22.35	-
GCWGC	2	m4C	24.17 %	3911	16,184	37.52	25.13	GCWGC
RGATCY	5	m4C	19.09 %	808	4,232	36.99	25.80	RGATCY
GGCC	3	m4C	18.02 %	3721	20,654	37.67	26.39	GGCC

#### CRISPR system

MinCED derived from the CRT [65], was used to predict CRISPR structure. CRISPR are extensively found in prokaryotes and are thought to compose a CRISPR-associated system, which is a putative immune system based on RNA-interference [66]. Three candidate CRISPR clusters on chromosome sequence were annotated under strict parameter and 1 CRISPR on plasmid (further information is available in Additional file 4).

### Genomic islands

**Fig. 6:**
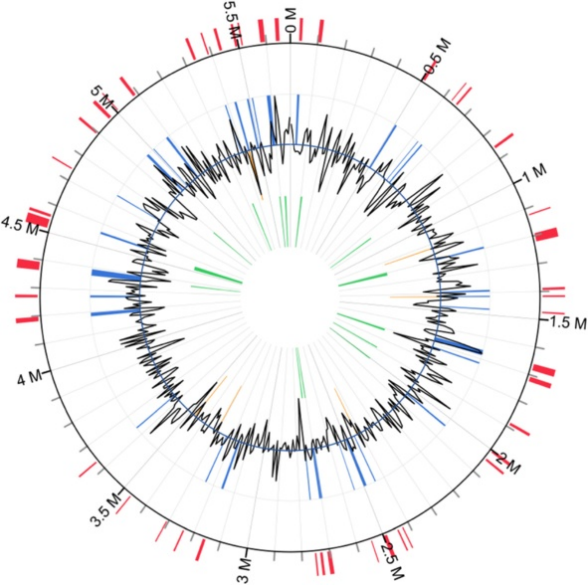
GEIs distribution in the chromosome of *M. panniformis* FACHB1757. From inside to outside, green bars illustrate IslandPick prediction, orange bars show the results annotated by SIGI-HMM, and blue bars are predicted by IslandPath-DIMOB. Red bars indicate integrated GEIs candidate positions. Black line plot around the small circle reveal the GC content

GEIs are particularly influential in microorganism genomes with regard to virulence, antibiotic resistance, metabolic, symbiosis, or other important adaptations [67]. GEIs have substantial roles in horizontal gene transfer, which is now widely acknowledged as an important force that shapes bacterial genome structure. Island Viewer (version 2.0) [68] was used to predict the GEIs in *M. panniformis*FACHB1757. Island Viewer integrates SIGI-HMM, Island Pick, and Island Path-DIMOB and built-in databases, including the Virulence Factor Database and Antibiotic Resistance Gene Database. Thirty-six GEIs were found using Island Viewer, and their positions are shown in Fig. 6. Different kinds of functions were identified and are summarized in Table 6. Transposases were identified in most of the GEIs, as they participated in horizontal gene transfer. Toxin-related gene clusters were annotated in six GEIs and probably affect competitiveness and fitness. Some functional genes, such as *hat*/*hatR*, were also detected, which indicates the enhanced adaptability and metabolic versatility in this strain.

**Table 6:** Functions and types of all 36 GEIs in chromosome

Function	Advantage conferred	GEI type	Related GEIs
Alkaline phosphatase	Increased metabolic versatility	Metabolic	GEI2,GEI7,GEI10,GEI17,GEI19,GEI23,GEI26,GEI27,
Toxin/Antitoxin protein	Competitiveness	Pathogenicity, resistance	GEI1,GEI6,GEI13,GEI31,GEI32,GEI33
Transferase	Increased metabolic versatility	Metabolic	GEI4,GEI9,GEI15,GEI21,GEI24,GEI25,GEI30,GEI36
Transposase	Increased metabolic versatility	Metabolic	GEI1,GEI3,GEI4,GEI11,GEI12,GEI15,GEI16,GEI18, GEI24,GEI29,GEI34
Hat/HatR	Increased metabolic versatility, increased adaptability	Fitness	GEI11,GEI28
Heat shock protein	Increased metabolic versatility, increased adaptability	Synthesis, fitness	GEI31
PsaE	Increased metabolic versatility	Metabolic, fitness	GEI9

## Conclusions

This study presents the complete whole genome sequence of a newly recorded species in China, *M. panniformis,* and demonstrates several genomic perspectives, including comparison with nine other water bloom-forming cyanobacterial species. A 5.6 Mbp chromosome with a 38 Kbp plasmid was reported, and gene function, methylation modification, CRISPR, and GEIs throughout the genome were described. Large-scale of structure variation was demonstrated by comparison with *M. aeruginosa* genomes. A Venn diagram of four *Microcystis* strains showed gene quantity and category variation as a result of evolutionary divergence and revealed that *Microcystis* has underwent flexible genome evolution.

## Additional files

Additional file 1: Tables S1-3 and Figures S1-5.
**Table S1.** General information regarding phylogenomics and comparative genomic analysis. **Table S2.** Function annotation assignment from different databases. **Table S3.** Conserved gene cluster function and position in the genomes of four *Microcystis* strains. **Figure S1.** Whole genome comparison with *M. aeruginosa* NIES843 at the nucleic acid level. Large-scale structural rearrangement was obvious at the nucleic acid level. Blocks on the line for *M. panniformis* FACHB1757 show that the sequences of the two species were in the same direction, whereas blocks under the line indicated that the sequences ran in the opposite direction. **Figure S2.** Length distribution of annotated protein-coding genes. The majority of fractions varied in length from 100 to 150. **Figure S3.** Subsystems and category distributions of genes. Functional annotation by RAST. **Figure S4.** Histogram demonstrating functional annotation results against GO. **Figure S5.** Number of each category in function annotation results against KEGG. (DOCX 599 kb) Additional file 2:
**The COG function annotation in each COG cluster.** (XLSX 114 kb) Additional file 3:
**N** ^**4**^ **-methylcytosine, N** ^**6**^ **-methyladenine, and other modified bases throughout the whole chromosome.** (XLSX 7893 kb) Additional file 4:
**Annotation of three candidate CRISPR clusters on the chromosome sequence and one CRISPR cluster on the plasmid using strict parameters.** (XLSX 14 kb)

## Abbreviations

CRISPR: Clustered Regularly Interspaced Short Palindromic Repeats
CRT: CRISPR Recognition Tool
EPS: Extracellular polysaccharide
GEIs: Genome islands
MinCED: Mining CRISPRs in Environmental Datasets
R–M: Restriction–modification
TRs: Tandem repeats
