# Supplementary material for: Complete genome sequence and genomic characterization of Microcystis panniformis FACHB 1757 by third-generation sequencing
Source: Stand Genomic Sci. 2016 Jan 28;11:11. doi: 10.1186/s40793-016-0130-5 (PMC4730716; doi:10.1186/s40793-016-0130-5)
Supplement: Additional file 1: Tables S1-3 and Figures S1-5. — Table S1. General information regarding phylogenomics and comparative genomic analysis. Table S2. Function annotation assignment from different databases. Table S3. Conserved gene cluster function and position in the genomes of four Microcystis strains. Figure S1. Whole genome comparison with M. aeruginosa NIES843 at the nucleic acid level. Large-scale structural rearrangement was obvious at the nucleic acid level. Blocks on the line for M. panniformis FACHB1757 show that the sequences of the two species were in the same direction, whereas blocks under the line indicated that the sequences ran in the opposite direction. Figure S2. Length distribution of annotated protein-coding genes. The majority of fractions varied in length from 100 to 150. Figure S3. Subsystems and category distributions of genes. Functional annotation by RAST. Figure S4. Histogram demonstrating functional annotation results against GO. Figure S5. Number of each category in function annotation results against KEGG. (DOCX 599 kb) [file 40793_2016_130_MOESM1_ESM.docx]

**Table S1 General information regarding phylogenomics and comparative genomic analysis**

| #Organism/Name | BioProject | Size (Mb) | GC% | Scaffolds | Genes | Proteins | Level |
| --- | --- | --- | --- | --- | --- | --- | --- |
| *Microcystis aeruginosa* NIES843 | PRJNA59101 | 5.84279 | 42.3 | 1 | 6364 | 6312 | CG^*^ |
| *Microcystis aeruginosa* NIES2549 | PRJNA224116 | 4.29421 | 42.9 | 1 | 3952 | 3834 | CG |
| *Microcystis aeruginosa* PCC7806 | PRJNA15702 | 5.17280 | 42 | 116 | 5383 | 5292 | Contig |
| *Anabaena* sp. 90 | PRJNA179383 | 5.30567 | 38.1 | 5 | 4673 | 4517 | CG |
| *Raphidiopsis brookii* D9 | PRJNA42981 | 3.18651 | 40.1 | 47 | 2927 | 2634 | Contig |
| *Pseudanabaena* sp. PCC7367 | PRJNA183004 | 4.88568 | 46.2 | 2 | 3967 | 3891 | CG |
| *Synechococcus elongatus* PCC6301 | PRJNA58235 | 2.69625 | 55.5 | 1 | 2664 | 2567 | CG |
| *Aphanizomenon flos-aquae* 2012/KM/D3 | SAMN02954153 | 5.74177 | 38.2 | 325 | 5478 | 4415 | Contig |
| *Planktothrix agardhii* NIVA-CYA 126/8 | PRJNA163669 | 5.04587 | 39.6 | 6 | 4259 | 4191 | C^**^ |
| *Cylindrospermopsis raciborskii* CS-505 | PRJNA42983 | 3.87903 | 40.2 | 93 | 3501 | 3449 | Contig |
| *Nodularia spumigena* CCY9414 | PRJNA199922 | 5.46527 | 41.2 | 1 | 4834 | 4754 | C |
| *Synechococcus elongatus* PCC7942 | PRJNA58045 | 2.74227 | 55.5 | 2 | 2719 | 2661 | CG |
| *Synechocystis* sp. PCC6803 | PRJNA57659 | 3.94702 | 47.3 | 5 | 3625 | 3575 | CG |

*CG: Complete genome **C: Chromosome

**Table S2 Function annotation assignment from different databases**

| Database | Assigned Number | Percent (%) |
| --- | --- | --- |
| COG | 3373 | 51.74% |
| GO | 2703 | 41.46% |
| KEGG | 897 | 13.76% |
| NR | 2167 | 33.24% |
| Uniprot | 2678 | 41.08% |
| Annotated | 3921 | 60.15% |
| Total | 6519 | 100.00% |

**Table S3 Conserved gene cluster function and position in the genomes of four *Microcystis* strains**

#: Not available


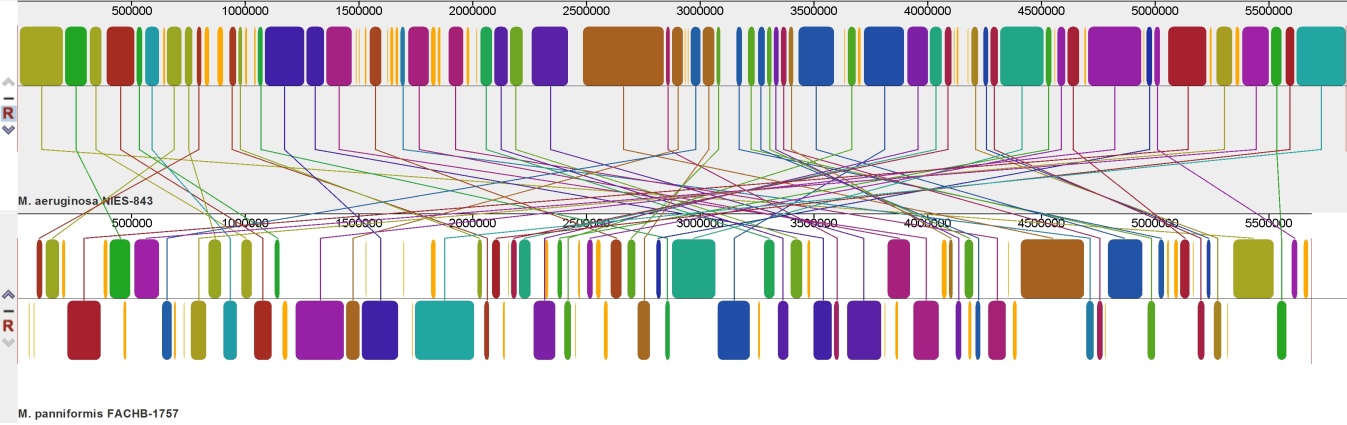


**Figure S1 Whole genome comparison with *M. aeruginosa* NIES843 at the nucleic acid level.**


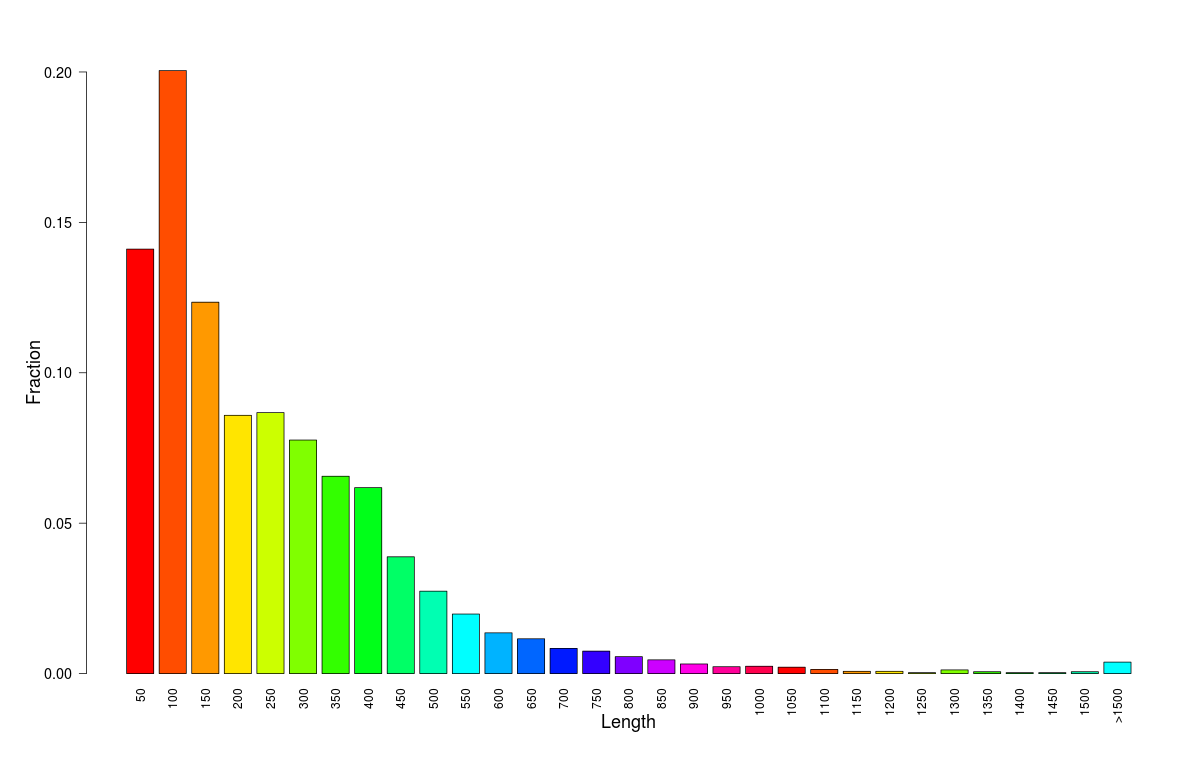
**Figure S2 Length distribution of annotated protein-coding genes.**

**
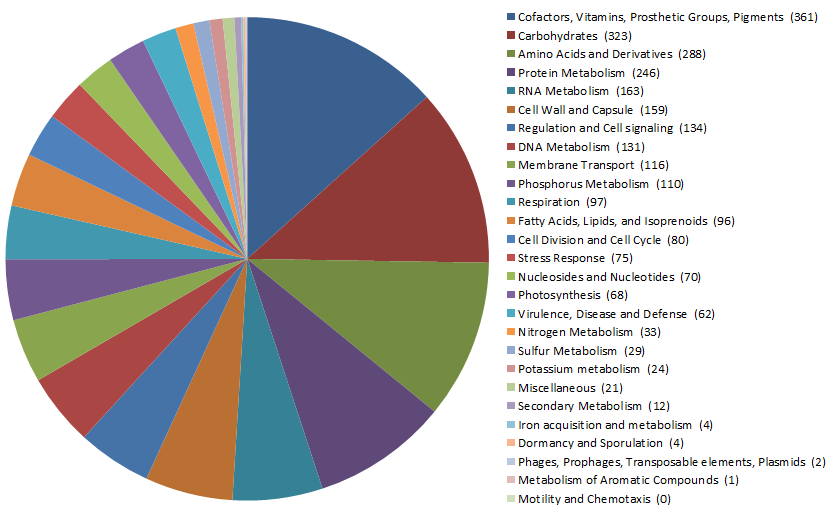
**

**Figure S3 Subsystems and category distributions of genes.**


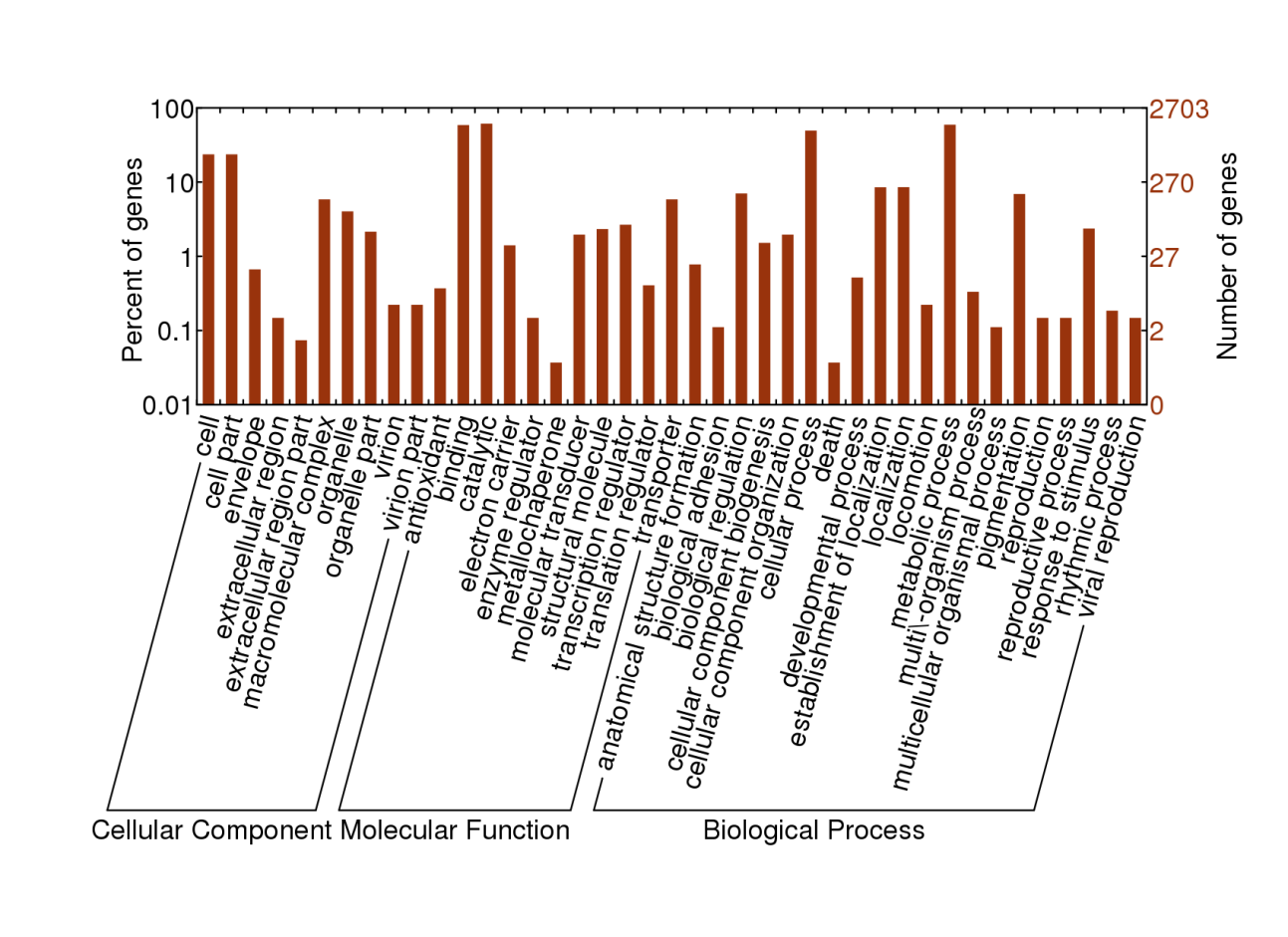


**Figure S4 Histogram demonstrating functional annotation results against GO.**


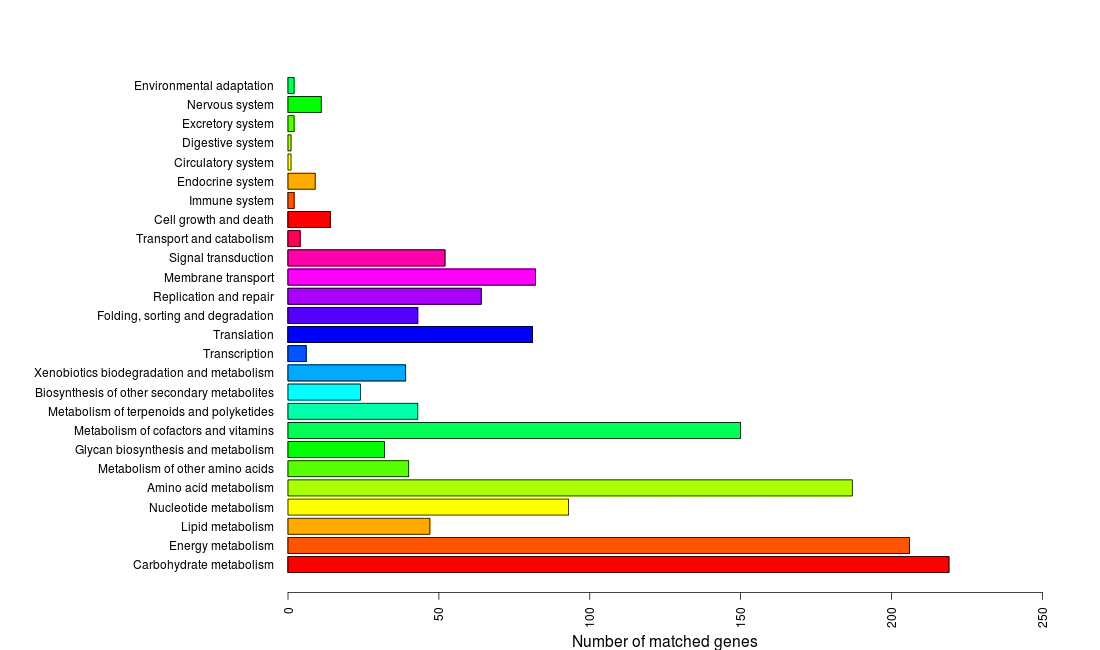
**Figure S5 Number of each category in function annotation results against KEGG.**
